# Chemoradiotherapy Increases Intratumor Heterogeneity of HPSE Expression in the Relapsed Glioblastoma Tumors

**DOI:** 10.3390/ijms21041301

**Published:** 2020-02-14

**Authors:** Anastasia V. Suhovskih, Galina M. Kazanskaya, Alexander M. Volkov, Alexandra Y. Tsidulko, Svetlana V. Aidagulova, Elvira V. Grigorieva

**Affiliations:** 1 Institute of Molecular Biology and Biophysics FRC FTM, 2/12, Timakova str., 630117 Novosibirsk, Russia; alexandra.tsidulko@gmail.com (A.Y.T.); elv_grig@yahoo.com (E.V.G.); 2 Novosibirsk State University, 1, Pirogova str., 630090 Novosibirsk, Russia; 3 Meshalkin National Medical Research Centre, 15, Rechkunovskaya str., 630055 Novosibirsk, Russia; g_kazanskaya@meshalkin.ru (G.M.K.); a_volkov@meshalkin.ru (A.M.V.); 4 Novosibirsk State Medical University, 52, Krasny Prospect, 630091 Novosibirsk, Russia; a_sv@ngs.ru

**Keywords:** glioblastoma multiforme, adjuvant chemoradiotherapy, disease relapse, heparan sulfate, heparanase expression, intratumor heterogeneity

## Abstract

Adjuvant chemoradiotherapy is a standard treatment option for glioblastoma multiforme (GBM). Despite intensive care, recurrent tumors developed during the first year are fatal for the patients. Possibly contributing to this effect, among other causes, is that therapy induces changes of polysaccharide heparan sulfate (HS) chains in the cancer cells and/or tumor microenvironment. The aim of this study was to perform a comparative analysis of heparanase (HPSE) expression and HS content in different normal and GBM brain tissues. Immunohistochemical analysis revealed a significant decrease of HPSE protein content in the tumor (12-15-fold) and paratumorous (2.5-3-fold) GBM tissues compared with normal brain tissue, both in cellular and extracellular compartments. The relapsed GBM tumors demonstrated significantly higher intertumor and/or intratumor heterogeneity of HPSE and HS content and distribution compared with the matched primary ones (from the same patient) (*n* = 8), although overall expression levels did not show significant differences, suggesting local deterioration of HPSE expression with reference to the control system or by the treatment. Double immunofluorescence staining of various glioblastoma cell lines (U87, U343, LN18, LN71, T406) demonstrated a complex pattern of HPSE expression and HS content with a tendency towards a negative association of these parameters. Taken together, the results demonstrate the increase of intratumor heterogeneity of HPSE protein in relapsed GBM tumors and suggest misbalance of HPSE expression regulation by the adjuvant anti-GBM chemoradiotherapy.

## 1. Introduction

Conventional anti-glioblastoma multiforme (GBM) therapy includes maximal surgical resection, followed by a combination of radiotherapy (60 Gy) and chemotherapy with temozolomide (TMZ) [1,2]. Despite the intensive treatment, about 70% of GBM patients develop disease relapse within one year of diagnosis [3]. Study of mechanisms underlying the development of relapse is an important way to overcome the problem and increase efficiency of the anti-GBM treatment. One possible cause of relapse may be therapy-induced disorders in the extracellular matrix (ECM) of brain tissue, hosting the residual GBM cells. Normal brain ECM mainly consists of hyaluronic acid and complex polysaccharide–protein molecules of proteoglycans (PGs) bearing polysaccharide chains of chondroitin sulfate and/or heparan sulfate (HS). These molecules are tightly involved in cell–cell and cell–matrix interactions as well as cell signaling, and their distortion by intensive chemoradiotherapy might be of importance for the residual GBM cells and their interaction with the surrounding brain tissue and relapse development. It was shown that HS is involved in glioma development, and its accumulation in GBM tumors is associated with low relapse-free survival for GBM patients [4].

HS degradation in the ECM and at the cell surface contributes to ECM remodeling and is an active process depending on heparanase (HPSE)—an endoglycosidase that cleaves heparan sulfate (HS) chains of heparan sulfate proteoglycans [5]. HS participates in cell signaling pathways and cell–microenvironment interactions [6] and plays an important role in brain development and glioma progression [7]. The enzyme is involved in the crosstalk between cells and their microenvironment due to HS degradation and ECM remodeling [8]. HPSE expression is increased in different cancers and is associated with aggressive disease and poor prognosis [9,10,11,12].

Information about HPSE expression in gliomas is controversial. It has been previously shown that HPSE mRNA is increased in oligodendroglioma (Grade II), anaplastic astrocytoma (Grade III) and glioblastoma (Grade IV) compared with normal brain tissue; Western blot has demonstrated the increase of HPSE protein in anaplastic astrocytoma and glioblastoma. In addition, the expression of HPSE is correlated with enhanced Ki-67 index in glioma tumors [13]. Immunohistochemistry IHC analysis has revealed low levels of HPSE in normal brain tissue and elevated levels in Grades II and IV; high HPSE expression in patients with glioblastoma was found to be associated with shorter survival [14]. One of the possible mechanisms by which HPSE promotes glioma progression could be an induction of CD24 that provides glioma cell invasion, migration and proliferation and promotes tumor growth [15]. However, according to other studies, HPSE expression was almost undetectable in GBM tumors and was not implicated in the invasiveness of GBM to surrounding healthy brain tissue in vivo [16]. In a xenograft tumor model, relatively moderate HPSE expression level significantly enhanced experimental U87 tumor development and tumor vascularity, whereas high expression level inhibited the tumor growth [17]. Changes in HPSE expression may affect HS, which is a key component of glioma microenvironment; the fine structure of HS chains plays an important role in cell–cell interactions, adhesion, migration and signaling [7,18].

Previously, we have shown that mRNA level of HPSE is significantly decreased in Grade II-IV gliomas [19] and distribution of its substrate HS is associated with low relapse-free survival of the glioma patients [4]. To study a possible effect of adjuvant anti-glioblastoma chemoradiotherapy on HPSE expression and its interrelation with HS content, we performed their comparative analysis in primary tumors and the relapsed tumors developed after the treatment.

## 2. Results

### 2.1. Chemoradiotherapy Affects Intertumor Heterogeneity of HPSE Content and Distribution

**Figure 1:**
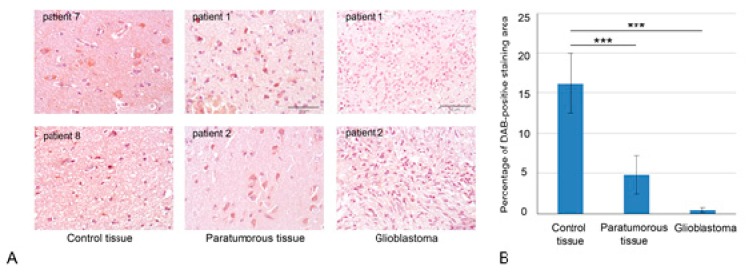
Heparanase (HPSE) expression in normal human brain tissues and GBM tumors. (**A**) Immunohistochemical analysis of HPSE expression in control brain tissue, paratumorous and GBM tissues. Representative pictures are shown. Magnification: 400×. Scale bars: 50 μm. (**B**) Quantitative analysis of anti-HPSE staining intensities in different brain tissues. Bars represent the mean for all the clinical samples for each tissue type from triplicate experiments ± SD (Origin 8.5). *** *p* < 0.001.

Previously, a significant decrease of mRNA levels of HPSE in Grade II-IV gliomas and heterogeneous distribution of HPSE protein in primary glioblastomas were demonstrated [19]. To investigate a hypothesis on a potential negative effect of anti-GBM treatment on HPSE and HS content and distribution in GBM tissues, we collected matched pairs of primary and relapsed GBM tumors from the same patient (*n* = 8). Quantitative analysis of HPSE protein content in normal human brain tissue (where the tumor and paratumorous tissues were matched pairs for each patient with malignancy, from the central part of the tumor and a more distant part of the brain) and primary and relapsed GBM tumors was performed based on IHC staining (Figure 1, Supplemental Figure S1).

HPSE protein content in the studied brain tissues was scored on a "three plus" basis for the percentage of stained cells and their intensity (ranging from “0” for no specific staining to “+++” for strong signal in >50% of cells or very strong signal in ECM). The highest HPSE content was shown in the morphologically normal human brain tissue (30%–35% "+" plus 5% "++"), whereas paratumorous (12%–15% "+" plus 2%–3% "++") and glioblastoma (almost no expression) brain tissues possessed significantly decreased staining intensity. Quantitative analysis of the DAB signal with ZENblue software (Zeiss, Germany) (Olympus) demonstrated 2.5–3-fold decrease of HPSE protein content in paratumorous brain tissue and 12–15-fold decrease in glioblastoma compared with the morphologically normal human brain tissue (both in cellular and extracellular compartments).

**Figure 2:**
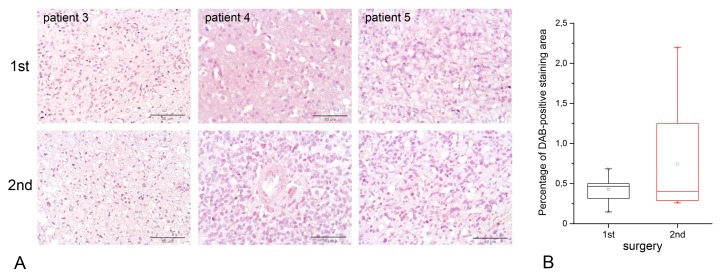
HPSE expression in primary glioblastoma (first surgery) and relapsed one (second surgery). (**A**) Immunohistochemical analysis of HPSE expression in primary and relapsed glioblastoma. Representative pictures are shown. Magnification: 400×. Scale bars: 50 μm. (**B**) Quantitative analysis of anti-HPSE staining intensities in primary and relapsed glioblastoma. Bars represent the mean for all the clinical samples for each tissue type from triplicate experiments ± SD (Origin 8.5; OriginLab Corporation, Northampton, USA).

Comparative analysis of HPSE content and distribution in the specimens obtained from the first surgery and second surgery (relapse) for the same patient revealed significantly higher heterogeneity of the HPSE expression level in the relapsed GBM tumors, whereas median HPSE expression levels in the samples were extremely low and formally demonstrated no statistically significant differences (Figure 2, Supplemental Figure S1).

### 2.2. Interrelation of HPSE and HS Content in GBM Tumors and Cell Lines

**Figure 3:**
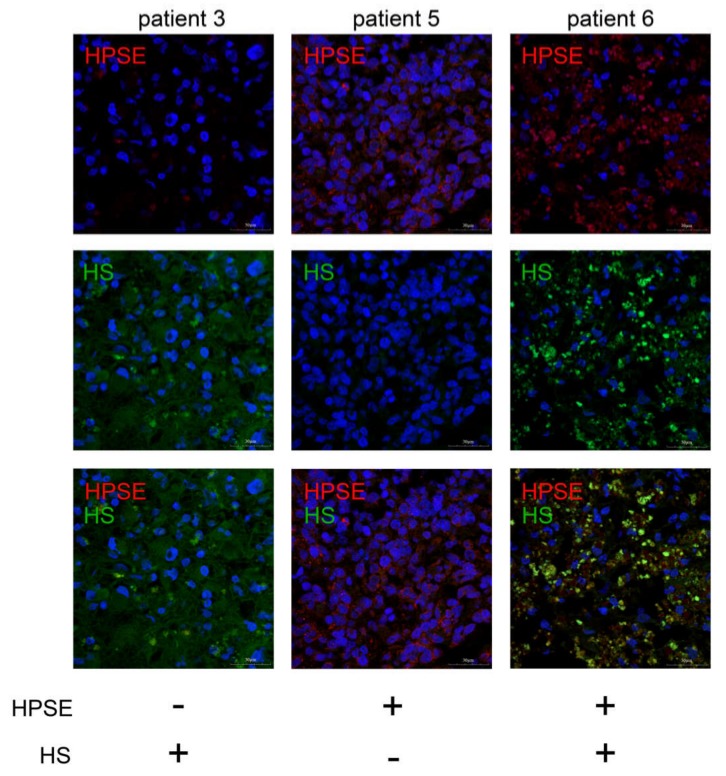
HPSE and heparan sulfate in glioblastoma tissues. Intertumor variability of HPSE expression and HS content in glioblastoma specimens. HPSE was visualized with Alexa 647-conjugated anti-rabbit antibody; HS was visualized with Alexa 488-conjugated anti-mouse antibody. Magnification: 400×. Scale bars: 50 μm.

To study the interrelation between HPSE expression and HS content in the same glioblastoma specimens, double immunostaining for HPSE and HS with different fluorescent probes was performed (Figure 3, Supplemental Figures S2 and S3).

Different combinations of these parameters were detected in GBM tumors—low HPSE expression/high HS content or high HPSE expression/low HS content or high HPSE expression/high HS content (representative pictures are presented). A common tendency for a negative association between the HPSE expression and HS content was observed, although the result was not statistically significant.

**Figure 4:**
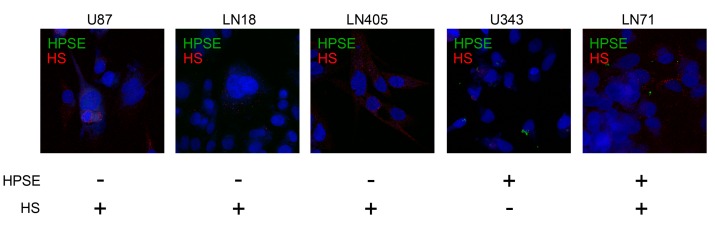
Immunofluorescence analysis of HPSE protein and HS content in different glioblastoma cell lines. Heparanase was visualized with Alexa 488-conjugated anti-rabbit antibody; HS was visualized with Alexa 647-conjugated anti-mouse antibody. The nuclei were counterstained with DAPI. Magnification: 400×.

To check a hypothesis that the observed high intratumor heterogeneity of HPSE protein and HS content in GBM tissues might result from heterogeneity of glioma cell subpopulations, different glioblastoma cell lines (U87, U343, LN18, LN71, LN405) were double-immunostained for HPSE and HS (Figure 4, Supplemental Figure S4).

HPSE expression was detected in U343 and LN71 cell lines only, whereas HS was present in all studied cell lines to various degrees, supporting the contribution of different glioma cell subtypes to intratumoral heterogeneity of HPSE protein and HS content in GBM tumors. 

Xenograft U87 tumors grown in brains of SCID mice retained low HPSE protein and high HS content corresponding to those in the inoculated U87 cells (Supplemental Figure S5).

## 3. Discussion

In this study, the obtained results showed that HPSE protein is down-regulated both in primary and recurrent GBM tissues with significantly elevated intertumor heterogeneity in the last ones. 

There is no consensus about HPSE expression in gliomas in the previously published data. HPSE transcription is up-regulated in glioma (Grade II-IV) compared with nonmalignant brain tissue [13].

Low levels of HPSE in normal brain tissue and elevated levels in Grades II and IV were demonstrated by IHC; high HPSE expression in patients with glioblastoma was found to be associated with shorter survival [14]. Expression of HPSE is also up-regulated in human pediatric central nervous system CNS embryonal cancers and medulloblastomas compared with healthy brain tissue, and treatment of pediatric brain tumor cells with HPSE inhibitors attenuates their growth [15]. In contrast, HPSE expression was not detected in GBM tumors and is not implicated in the invasiveness of GBM cells to surrounding healthy brain tissue in vivo [16]. Overexpression of HPSE in U87 glioma cells increases their invasion [17], and in U251n also increased cell invasion, proliferation and anchorage-independent colony formation [20]. We have previously shown that HPSE is down-regulated in gliomas Grade II–IV at the mRNA level, and there is a significant decrease of HPSE protein in tumor and paratumorous glioblastoma tissues compared with the normal tissue [19]. 

Such contradictory data can be due to the fact that the structure of HPSE is complex, and alternatively spliced forms of this enzyme are exist. The splice variant in the untranslated regions of the gene was described by Dong et al. [21]. Splicing variants of the HPSE gene have different effects on trafficking, processing of the HPSE protein and loss of its enzymatic activity [22,23]. Two splice variants—XHpaS and XHpaL from *Xenopus laevis*—both interact with heparin and HS; however, they have different enzymatic activity, and over-expression of XHpaS but not XHpaL increases cell adhesion of glioma cells to HS-coated surfaces [24]. Splice variant 36 of heparanase from *Spalax* has anti-glioma activity that inhibits HS degradation, suppresses glioma tumor growth and decreases experimental B16-BL6 lung colonization in a mouse model. On the contrary, *Spalax* splice variant 7 of HPSE is devoid of enzymatic activity but enhances tumor growth [25]. Furthermore, HPSE expression level can be associated with tumor development differently: moderate HPSE expression significantly enhances tumor development and tumor vascularity, whereas high expression level inhibits the tumor growth [17].

It is known that HPSE expression is regulated through multiple epigenetic mechanisms including histone modifications [26], promoter DNA methylation [27], transcriptional factors [28], sequences at the 3′ untranslated region (3′ UTR) of the gene [29] and enhancer RNA [30]. Possibly, some of these mechanisms are destroyed in relapsed GBM tumors after adjuvant anti-GBM chemoradiotherapy; therefore, significant variability in the HPSE expression is present.

The interrelation of HPSE expression with the presence of its substrate HS was not studied yet, although some data on HS presence in gliomas are available. It was shown that glioblastoma Grade IV possesses higher HS than astrocytoma Grade II [31]; HS content is present in patient-derived glioma tumor sphere cell lines and within the same tumor in a highly heterogeneous manner [32]. Increased HS content is demonstrated in GBM tumors and associated with low relapse-free survival of the GBM patients [4]. The obtained data on the decreased expression of HPSE in GBM tumors and the tendency of its negative correlation with HS content might suggest a possible molecular mechanism for HS upregulation in GBM tumors, along with other mechanisms involved in maintaining HS content in brain tissue. 

## 4. Materials and Methods

### 4.1. Patients and Tissue Samples

All tissue samples were obtained from primary and relapse GBM tumors (matched pair for each patient in the study) during radical surgery at the Neurosurgical Unit of Meshalkin National Medical Research Center (Novosibirsk, Russia). Tissues were fixed in 10% formalin solution and embedded in paraffin. Haematoxylin & Eosin H&E-stained sections were prepared to define representative tumor regions, and GBM diagnosis was confirmed according to the World Health Organization (WHO) classification by qualified pathologists. In total, 16 tissue samples from eight patients with diagnosis of glioblastoma (WHO Grade IV) (matched pairs—from first and second surgeries) were used in the study. Brain tissue from patients with vascular malformation was used as control tissue.

The patient sample group had a mean and median age of 50.5 years. All the patients received adjuvant therapy (temozolomide, 5 days/month repeating courses, in combination with radiotherapy, 30 days, 2 Gy/day, 60 Gy in total). All procedures performed in the study involving human participants were in accordance with the ethical standards of the IMBB FRC FTM ethical committee and with the 1964 Helsinki Declaration and its later amendments or comparable ethical standards. Informed consent was obtained for all individual participants included in the study. Clinical information is presented in Table 1.

### 4.2. Immunostaining

For immunohistochemistry, 3- to 4-μm sections of formalin-fixed, paraffin-embedded tissue were deparaffinized, and antigen was retrieved by treatment with sodium citrate buffer (10 mM sodium citrate, 0.05% Tween-20) at 95–98 °C for 20 min. The rabbit polyclonal anti-HPSE (Abcam, cat. N ab42817,1:100) and mouse monoclonal anti-heparan sulfate (Millipore, cat. N MAB2040, 1:100) primary antibodies were used for immunostaining. Staining patterns for HPSE were visualized with Histostain-Plus 3rd Gen IHC Detection Kit (ThermoFisher Scientific, Waltham, MA, USA). The sections were counterstained with hematoxylin and observed by light microscopy using an AxioScopeA1 microscope (Zeiss, Oberkochen, Germany). Quantitative analysis was performed with ZENblue program (Zeiss). For immunofluorescent analysis, HPSE and HS were visualized with Alexa 647-conjugated anti-rabbit antibody (ThermoFisher Scientific, cat. N ab150063, 1:1000) and Alexa 488-conjugated anti-mouse (Abcam, cat. N ab150117, 1:1000) secondary antibodies, respectively. The slides were mounted and counterstained with DAPI using Prolong Gold SlowFade with DAPI mounting medium (ThermoFisher Scientific, Waltham, MA, USA) and observed by confocal laser scanning biological microscope Fluoview FV1000 (Olympus, Japan) with FV10-ASW software (Olympus). Seven tissue sections for each clinical sample were stained and analyzed. The staining analysis was performed by two qualified pathologists independently on a "three plus" basis for the percentage of stained cells and their intensity (0 was assigned to the samples with nondetectable expression, + and ++ to low and medium expression, respectively, and +++ to maximal expression, strong staining in > 50% of cells). Ten fields for each section were counted.

### 4.3. Immunocytochemistry

For immunocytochemical analysis, glioma cells (U87, U343, LN18, LN71, LN405) were grown on glass coverslips and then fixed with phosphate-buffered 4% formaldehyde. Mouse monoclonal anti-heparan sulfate (Millipore, cat. N MAB2040, 1:100) and rabbit polyclonal anti-heparanase (Abcam, cat. N ab42817, 1:100) were used for immunostaining. Staining patterns were visualized with Alexa 488-conjugated goat anti-rabbit IgG (ThermoFisher Scientific, cat N ab150077, 1:1000) and Alexa 647-conjugated goat anti-mouse IgG (Abcam, cat N ab150115, 1:1000) antibodies. The cells were mounted and counterstained with DAPI using Prolong Gold SlowFade Gold with DAPI mounting medium (Thermo Fisher Scientific, Waltham, MA, USA) and observed by confocal laser scanning biological microscope Fluoview FV1000 (Olympus, Japan).

### 4.4. Statistical Analysis

Statistical analyses were performed using Origin 8.5 software (OriginLab Corporation, Northampton, MA, USA); a value of *p* < 0.05 was considered to indicate a statistically significant difference. Data are expressed as the means ± SD.

## 5. Conclusions

We have demonstrated that HPSE expression is significantly decreased in paratumorous tissue compared with normal brain tissue, with a further decrease of this parameter in GBM tumors. These results suggest that HPSE expression levels in paratumorous tissue might be a potential molecular marker to delineate boundaries of pathological brain tissue to achieve more effective resection of the affected areas during surgery.

Relapsed tumors possess higher intertumor heterogeneity in HPSE content and distribution indicating a disturbance of a molecular mechanism for HPSE regulation by adjuvant chemoradiotherapy, which the GBM patients underwent between the first and second (relapse) surgeries. The observation is supported by cell line-specific patterns of HPSE and HS content in GBM cells as well. Overall, the data contribute to the brain extracellular matrix modulation by anti-glioblastoma treatment and understanding of pathophysiological mechanisms of glioblastoma relapsed tumor development.

## Figures and Tables

**Table 1:** Clinical characteristics of patients with glioblastoma.

Factors	N (patients) = 8	%
**Age**		
Median (range)	50.5 (38–68)	
> 50 median age	4	50
< 50 median age	4	50
**Sex**		
Male	4	50
Female	4	50
**Tumor development**		
Primary	5	62.5
Secondary	3	37.5
**Death**		
Yes	7	87.5
No	1	12.5
**IDH status**		
wild	8	100
mutant	0	0

## Supplementary Materials

The following are available online at https://www.mdpi.com/1422-0067/21/4/1301/s1.

## Abbreviations

GBM	Glioblastoma multiforme
ECM	Extracellular Matrix
Heparanase	HPSE
HS	Heparan Sulfate
