# Supplementary figures and images for: Chemoradiotherapy Increases Intratumor Heterogeneity of HPSE Expression in the Relapsed Glioblastoma Tumors
Source: Int J Mol Sci. 2020 Feb 14;21(4):1301. doi: 10.3390/ijms21041301 (PMC7073003; doi:10.3390/ijms21041301)

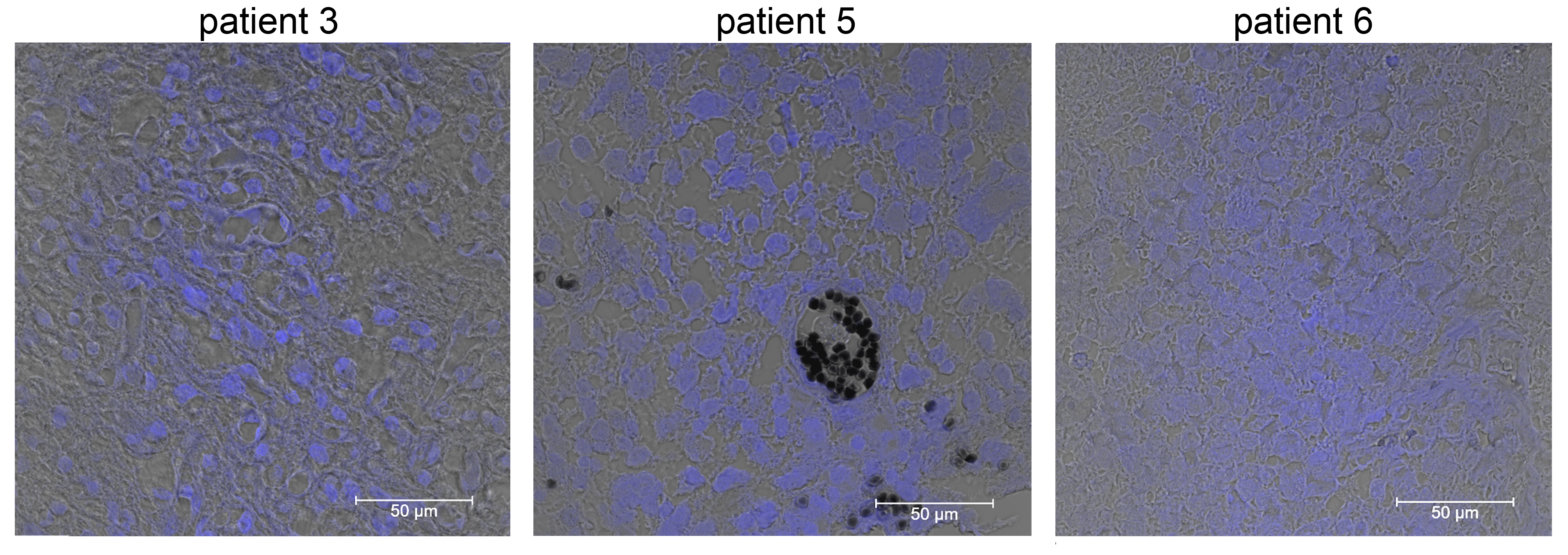

Supplement: Supplementary file 1 [file ijms-21-01301-s001.zip › Supplemental Figure 3.tif]

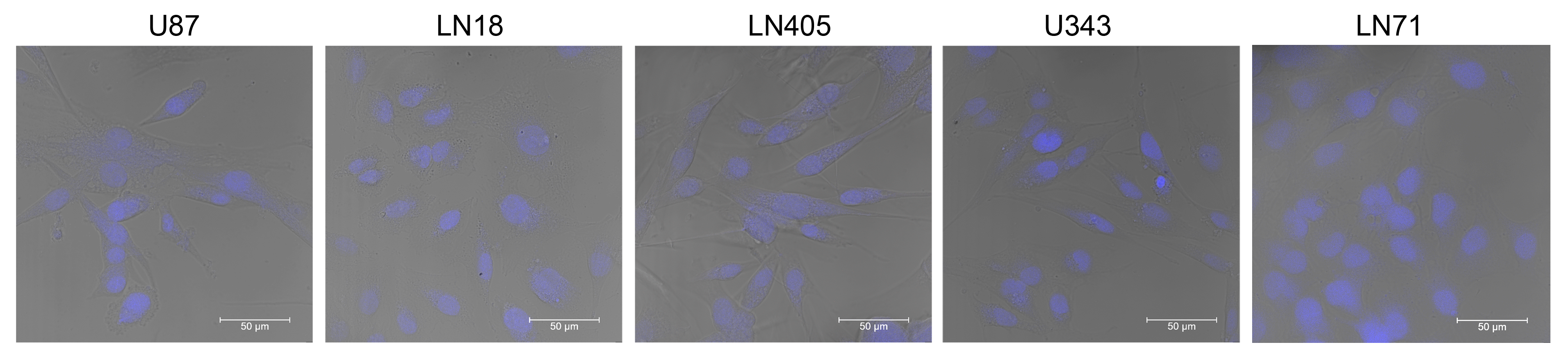

Supplement: Supplementary file 1 [file ijms-21-01301-s001.zip › Supplemental Figure 4.tif]

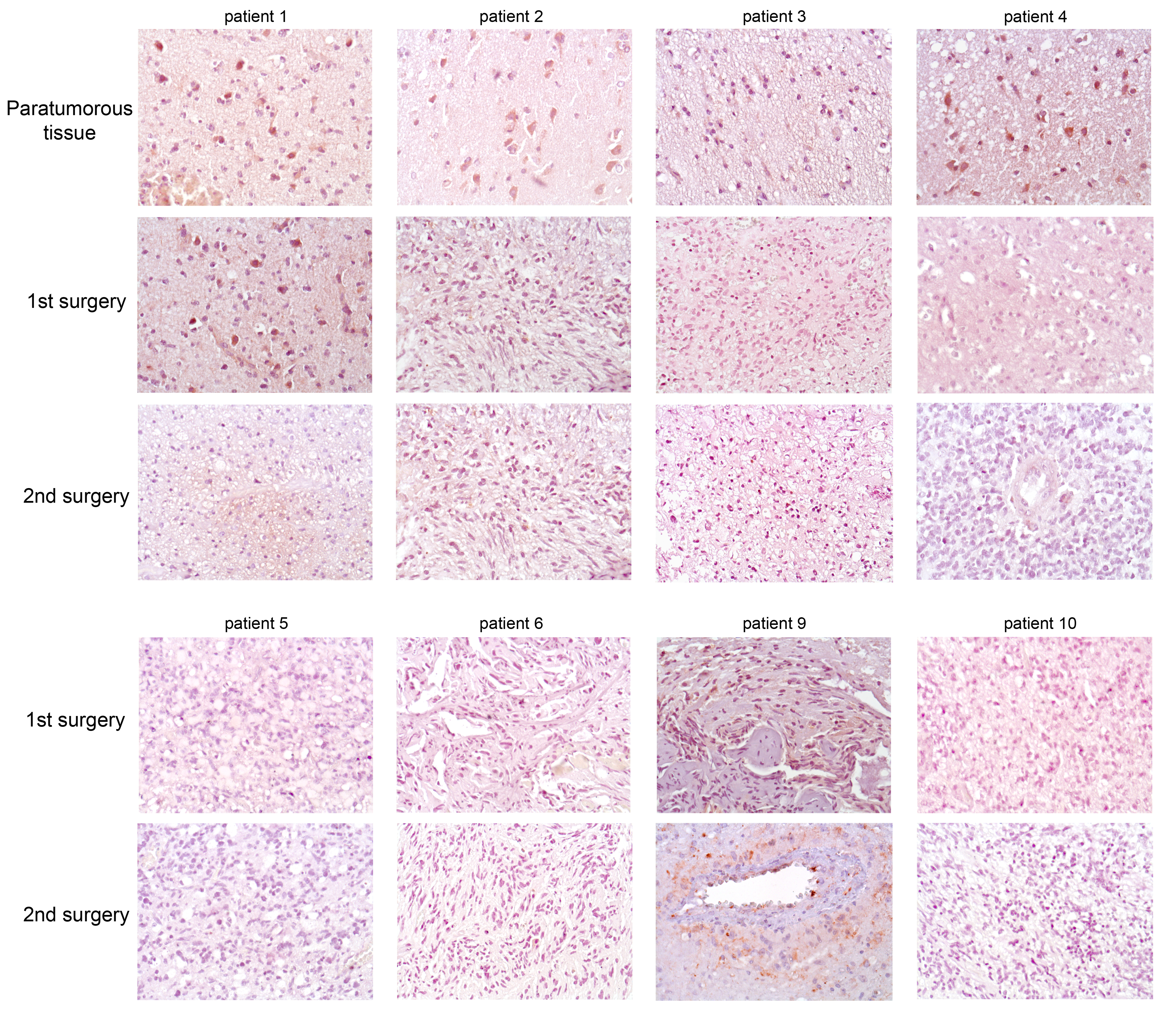

Supplement: Supplementary file 1 [file ijms-21-01301-s001.zip › Supplemental Figure 1.tif]

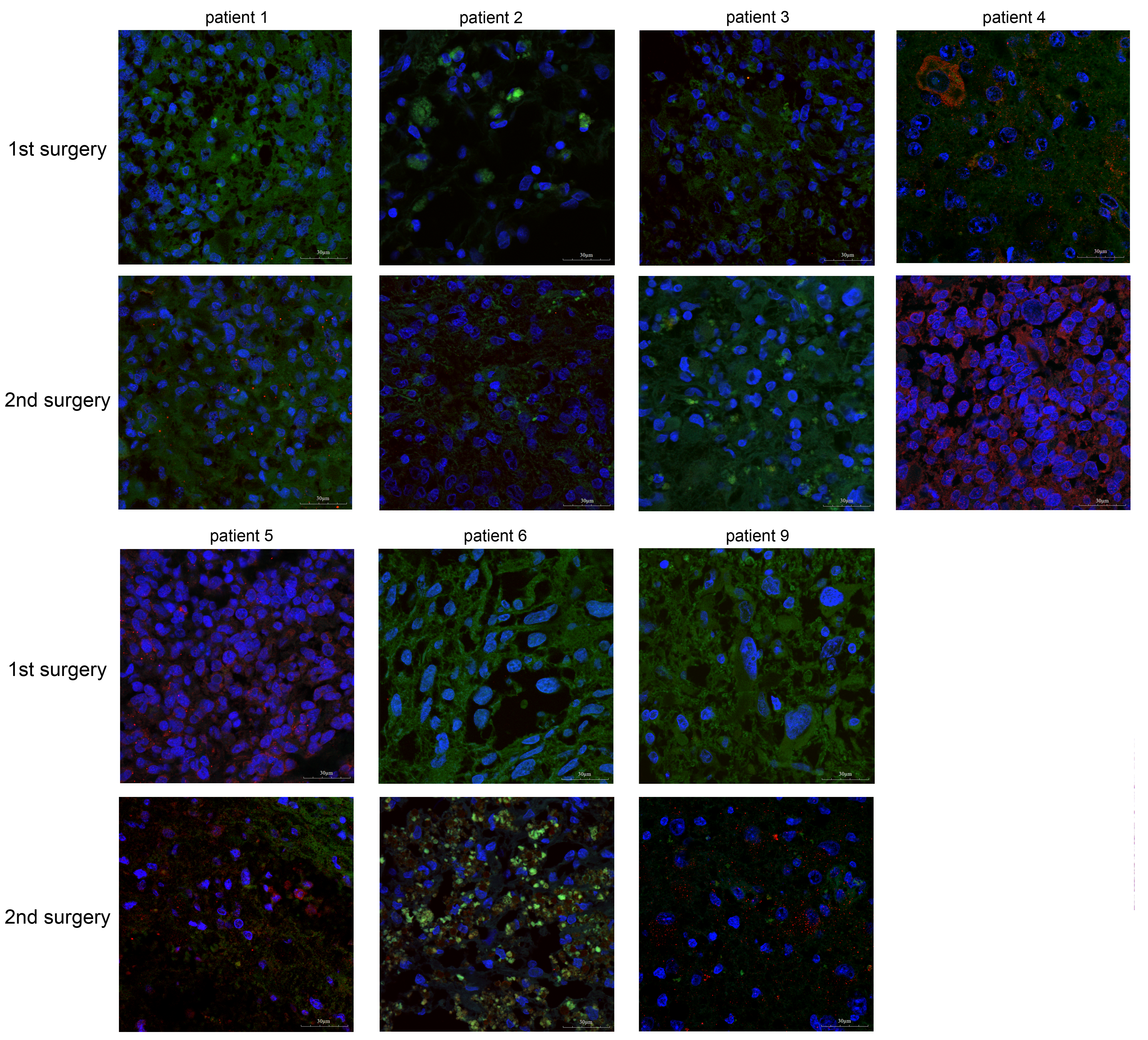

Supplement: Supplementary file 1 [file ijms-21-01301-s001.zip › Supplemental Figure 2.tif]
